# Hypoxia preconditioned serum (HPS) and platelet-rich plasma (PRP) promote proliferation, neurite network formation, and neurite elaboration in N2a cells

**DOI:** 10.3389/fbioe.2026.1901884

**Published:** 2026-09-04

**Authors:** Jun Jiang, Donya Safa, Samuel Knoedler, Jannat Altammar, Florian Falkner, Marc Hanschen, Haydar Kükrek, Ulf Dornseifer, Arndt F. Schilling, Hans-Günther Machens, Philipp Moog

**Affiliations:** 1 Experimental Plastic Surgery, Department for Plastic, Reconstructive and Hand Surgery, TUM University Hospital, Rechts der Isar, Munich, Germany; 2 Department of Plastic, Reconstructive and Aesthetic Surgery, ISAR Klinikum, Munich, Germany; 3 Division of Plastic Surgery, University Health Network, University of Toronto, Toronto, ON, Canada; 4 Department for Plastic, Hand, and Reconstructive Surgery, University Hospital Regensburg, Regensburg, Germany; 5 Department of Trauma Surgery, TUM University Hospital, Rechts der Isar, Munich, Germany; 6 Department of Trauma Surgery, Orthopedics and Plastic Surgery, University Medical Center Göttingen, Göttingen, Germany

**Keywords:** HPS, N2a neuroblastoma cells, neurite outgrowth, neurotrophic factors, PRP

## Abstract

**Background:**

The use of autologous growth factors, such as those contained in Hypoxia-Preconditioned Serum (HPS) and Platelet-Rich Plasma (PRP), represents a promising strategy to enhance regenerative processes in injured nerves. As an initial *in vitro* study, we investigated the effects of these secretomes on the neuroblastoma cell line N2a.

**Methods:**

HPS and PRP were first compared with normal serum (NS) using a protein microarray to detect neurologically related growth factors. Subsequently, N2a cells were cultured with three different secretome concentrations (0.1%, 1%, and 10%) for up to 96 h and analyzed for proliferation (cell counting), viability (Alamar Blue), cytotoxicity (LDH assay), combined proliferation and migration (scratch assay), and neurite outgrowth.

**Results:**

Microarray analysis identified 30 neurotrophic, regulatory, and inflammatory factors in both HPS and PRP. Compared with PRP, HPS contained significantly higher levels of BDNF, CNTF, and VEGF-A, as well as the regulatory factors IL-6, TGF-β, and LIF. Several neurodegeneration-associated markers, including FAS, TNF-α, and IL-8, were also elevated in HPS. Proliferation and migration were most strongly stimulated by the 1% concentration of HPS and PRP, which also maintained high viability and low cytotoxicity. After 96 h, both PRP and HPS markedly enhanced neurite outgrowth by increasing total neurite count, neurite length, neurite-covered area, and branching points. PRP-0.1% and -1% produced the strongest effects, with up to 1.7-fold higher values across assessed parameters than the negative control, whereas HPS-0.1% increased these parameters by up to 1.4-fold. Further analyses demonstrated that these effects occurred alongside marked cell proliferation. Although the proportion of neurite-bearing cells (NBCs) declined over time because total cell expansion outpaced the increase in NBCs, normalization of neurite parameters to NBCs revealed up to 1.7-fold increases in neurite count, neurite length, neurite-covered area, and branching points per NBC, with the strongest effects observed in HPS-0.1% and PRP-0.1%.

**Conclusion:**

HPS and PRP promoted expansion of the neurite network while enhancing neurite elaboration of NBCs despite pronounced proliferative effects. These findings identify HPS and PRP as biologically active secretomes with potential to support neuronal structural remodeling, warranting investigation in primary neuronal cultures and *in vivo* models of peripheral nerve regeneration.

## 1 Introduction

Peripheral nerve injuries remain a significant reconstructive challenge, often leading to incomplete functional recovery despite advanced surgical techniques (Houschyar et al., 2016). Although peripheral nerves have a greater regenerative potential than the central nervous system, full functional recovery is uncommon and often hampered by misdirected axonal growth or the development of neuropathic pain (Grinsell and Keating, 2014). Favorable outcomes are generally restricted to minor injuries such as neurapraxia or axonotmesis, whereas transected nerves require microsurgical repair due to the absence of spontaneous recovery (Lopes et al., 2022). Even with timely surgical intervention, regeneration proceeds slowly at a rate of approximately 1 mm per day in humans, leaving distal targets denervated for extended periods (Lopes et al., 2022). Furthermore, age-related structural and biochemical changes in the peripheral nervous system further diminish regenerative capacity, contributing to poorer outcomes in elderly patients (Porche et al., 2025).

Traditional strategies to improve nerve regeneration include microsurgical nerve repair, nerve grafting, and engineered biomaterials, but these approaches alone often fail to restore full function (Grosu-Bularda et al., 2025). One important biomaterial-based strategy is the use of nerve conduits, tubular structures designed to bridge nerve gaps and provide physical guidance for regenerating axons (Grosu-Bularda et al., 2025). While simple hollow conduits offer structural support, recent research has focused on enhanced conduits that incorporate biological and molecular cues to actively promote regeneration: For instance, conduits coated or loaded with neurotrophic factors such as nerve growth factor (NGF), brain-derived neurotrophic factor (BDNF), or glial cell line-derived neurotrophic factor (GDNF) have been shown to accelerate axonal sprouting and improve remyelination (Madduri et al., 2010; Su et al., 2020; Yang et al., 2011). Incorporating extracellular matrix components, Schwann cells, or stem-cell–derived exosomes into the conduit further augments regeneration by sustaining trophic support and cellular interactions (Guo et al., 2024; Yang et al., 2021; Jiang M. et al., 2024). In addition, biomaterial modifications such as nanofiber scaffolds or hydrogel coatings allow for controlled release of these agents, ensuring their prolonged availability at the injury site (Taisescu et al., 2025; Behtaj et al., 2022; Leung et al., 2022). These multifunctional conduits therefore combine structural guidance with targeted biochemical signals, representing a promising adjunct to conventional surgical repair.

In this context, biologically active therapies, such as autologous growth factor applications, have gained increasing attention as adjuncts to enhance nerve regeneration (Fakhr et al., 2024). Among these approaches, autologous growth factors derived from the patient’s own blood, such as Hypoxia Preconditioned Serum (HPS) and Platelet-rich Plasma (PRP), have emerged as promising candidates. These biological therapies provide a rich source of regenerative factors, including vascular endothelial growth factor (VEGF), platelet-derived growth factor (PDGF), basic fibroblast growth factor (bFGF), and insulin-like growth factor 1 (IGF-1) (Pavlovic et al., 2016; Jiang et al., 2023a; Jiang et al., 2023b). Of the two, PRP has been widely investigated in both experimental and clinical studies of peripheral nerve injury: Preclinical models have demonstrated that local application of PRP enhances axonal regrowth, promotes remyelination by activating Schwann cells and providing a sustained source of neurotrophic and angiogenic factors (Wang et al., 2022; Li et al., 2020; Pandunugrahadi et al., 2022). Clinically, PRP has been applied as an adjunct to microsurgical nerve repair or nerve grafting, with several reports suggesting improved sensory recovery and reduced neuropathic pain (Wang et al., 2024). These effects are thought to arise from PRP’s capacity to modulate the inflammatory response while simultaneously creating a pro-regenerative microenvironment at the injury site.

In contrast, HPS has not yet been studied in the context of peripheral nerve regeneration. HPS is generated by exposing peripheral blood cells to hypoxic conditions, which stimulates them to secrete a broad spectrum of regenerative cytokines and growth factors (Hadjipanayi and Schilling, 2013). This mechanism differs fundamentally from PRP, which is obtained by concentrating platelets and relying on platelet-derived growth factors upon activation. While its direct role in nerve regeneration remains unexplored, HPS has shown promising results in other regenerative applications, including (lymph-)angiogenesis (Jiang et al., 2023b; Moog et al., 2020), wound healing (Jiang et al., 2022a; Jiang et al., 2024b; Ektoras et al., 2023; Hadjipanayi et al., 2019), cartilage (Jiang et al., 2023a) and bone repair (Jiang et al., 2022b; Jiang et al., 2024c), and fat cell survival (Jiang et al., 2025). By mimicking the hypoxic microenvironment and inducing the upregulation of regenerative cytokines of all peripheral blood cells, HPS may offer a potent and alternative approach to PRP.

The application of these autologous growth factors may represent a patient-specific, minimally invasive, and biocompatible strategy to enhance nerve regeneration. As an initial *in vitro* study, we sought to provide the first basic evidence for possible neurogenic and neuritogenic effects of HPS and PRP on N2a neuroblastoma cells by assessing proliferation, viability, migration, and neurite outgrowth.

## 2 Materials and methods

### 2.1 Ethical approval

This study was conducted as per the Declaration of Helsinki and the approval of the ethics committee of the Technical University of Munich, Germany (File No.: 2025-571-S-CB; date of approval: 07 January 2026). Informed consent was obtained from all blood donors involved.

### 2.2 Production of hypoxia preconditioned serum (HPS)

HPS production was conducted according to an established protocol previously developed by our research group (Moog et al., 2020; Jiang et al., 2025). Blood samples were obtained from five healthy adult donors: Two female and three male; age range, 21–38 years. Donors were screened using predefined inclusion and exclusion criteria to reduce biological variability and exclude systemic inflammatory conditions: Individuals were excluded if they smoked, were pregnant, had systemic inflammatory disorders, or had used oral medication within the preceding 6 weeks. No additional donor typing was performed, because the study used pooled autologous secretomes for *in vitro* cell experiments rather than as a clinical allogeneic product. For HPS generation, 20 mL of peripheral venous blood was collected into a 30-mL syringe (Omnifix®, B. Braun AG, Melsungen, Germany). Thereafter, 5 mL of air was introduced through a 0.2-µm filter (Sterifix®, B. Braun AG, Melsungen, Germany), and the syringe was sealed. Samples were incubated for 4 days at 37 °C in a humidified atmosphere containing 5% CO_2_, during which physiological blood coagulation occurred while pericellular hypoxia of approximately 1% O_2_ developed as a consequence of oxygen consumption by peripheral blood cells. Following incubation, coagulation, and passive sedimentation, three distinct layers formed: The upper transparent fraction, which represents the HPS, was carefully collected through a 0.2-µm filter (Sterifix®, B. Braun AG, Melsungen, Germany) into a fresh syringe and stored individually at −80 °C for up to 3 months until use.

### 2.3 Production of normal serum (NS)

Normal serum (NS) was collected from the same donor cohort and served as a baseline serum control. Under sterile conditions, 20 mL of peripheral venous blood was drawn into separate 30-mL polypropylene syringes (Omnifix®, B. Braun AG, Melsungen, Germany). To obtain serum, the syringes were kept upright at room temperature (approximately 22 °C) for 4 h, allowing passive separation of blood components by sedimentation. The resulting serum supernatant was then transferred through a 0.2-µm filter (Sterifix®, B. Braun AG, Melsungen, Germany) into a fresh syringe. NS samples were stored individually at −80 °C for up to 3 months until use.

### 2.4 Production of platelet-rich plasma (PRP)

Platelet-rich plasma (PRP) was prepared from the same donor cohort using a standardized double-centrifugation protocol (Nagata et al., 2010). Briefly, 6 mL of peripheral venous blood was collected into trisodium citrate-containing blood collection tubes (366575, BD Vacutainer, Becton, Dickinson and Company, Franklin Lakes, NJ, USA) and centrifuged at 1,300 × *g* for 20 min. This first centrifugation step separated the blood into three layers: platelet-poor plasma in the upper fraction, a buffy coat containing platelets and leukocytes in the intermediate fraction, and erythrocytes in the lower fraction. The upper plasma layer together with the buffy coat, corresponding to approximately 60% of the total blood volume, was carefully transferred into a new tube. To reduce platelet loss, a small amount of the erythrocyte layer immediately beneath the buffy coat was also included. A second centrifugation step was then performed at 1,800 × *g* for 15 min, resulting in separation of the lower PRP fraction, approximately 0.5 mL, from the upper platelet-poor plasma fraction. The upper fraction was removed, and the remaining PRP was activated by adding 0.5 mL of thrombin (1 IU/mL; Tisseel, Baxter, IL, USA) and CaCl_2_ (8.88 μg/mL; Tisseel, Baxter, IL, USA) dissolved in basal medium (see 2.5). After incubation for 30 min at 37 °C, samples were centrifuged again at 2,500 × *g* for 20 min to obtain the activated PRP supernatant, corresponding to the PRP releasate dissolved in basal medium. The PRP releasate was collected using a sterile syringe, filtered through a 0.2-µm Sterifix® filter (B. Braun AG, Melsungen, Germany), and stored individually at −80 °C for up to 3 months before experimental use.

Validation of the preparation protocol was performed by comparing platelet concentrations in PRP with those in whole-blood samples using a C-Chip hemocytometer (DHC-N01, NanoEnTek, Korea). PRP and whole blood were diluted in PBS at ratios of 1:400 and 1:200, respectively. Subsequently, 10 µL of each diluted sample was loaded into the hemocytometer chambers, and platelets were manually counted using an inverted phase-contrast microscope (Axio Vert. A1, Carl Zeiss, Jena, Germany). Platelet concentrations were calculated according to the manufacturer’s instructions. PRP samples reached a mean platelet concentration of 1,235.43 ± 167.52 × 10^9^/L, compared with 225.34 ± 23.29 × 10^9^/L in whole blood.

### 2.5 Neuro-array–based analysis of neuronal growth factors in blood derivatives

To investigate the neurogenic potential of NS, HPS, and PRP, cytokine profiles were analyzed using a Human Neuro Discovery Array (RayBiotech, Peachtree Corners, GA, USA), which enables the detection of a total of 30 human proteins. Semiquantitative cytokine analysis was performed according to the manufacturer’s instructions. Signal intensity was recorded using a chemiluminescence imaging system (Mithras LB 940, Berthold Technologies GmbH & Co. KG, Bad Wildbad, Germany).

### 2.6 Cell culture

The murine neuroblastoma cell line N2a was cultured in Minimum Essential Medium (MEM) (PAN BIOTECH, Aidenbach, Germany) supplemented with 10% FCS (Biochrom GmbH, Berlin, Germany) and 1% Antibiotic/Antimycotic Solution (ab/am) (Antibiotic/Antimycotic Solution, Amphotericin B 25.00 μg/mL, Penicillin 10,000 Units/mL, Streptomycin 10.00 mg/mL, Capricorn Scientific GmbH, Ebsdorfergrund, Germany) at 37 °C in a humidified atmosphere containing 5% CO_2_ and used for subsequent experiments from passage 3 onward.

The basal medium consisted of MEM supplemented with 3% FCS and 1% ab/am and served as the negative control.

The experimental media consisted of HPS, PRP, and NS at concentrations of 0.1%, 1%, and 10%, each diluted (v/v) in basal medium. The individual HPS, PRP, and NS samples were pooled for the use in the cell experiments. Nerve growth factor (NGF-ß human, N1408, Sigma-Aldrich, St. Louis, Missouri, United States) was used as a positive control at 100 ng/mL in basal medium, a concentration commonly used to induce neurite outgrowth in N2a assays (Sindeeva et al., 2020).

### 2.7 Assessment of proliferation, viability, and cytotoxicity

For proliferation, viability, and cytotoxicity assessment of the test media, 5,000 cells were seeded per well in 24-well plates in 1,000 µL MEM supplemented with 10% FCS and incubated overnight. On the following day, the medium was replaced with the respective experimental media (HPS, NS and PRP at 0.1%, 1%, and 10%), as well as positive and negative control media (1,000 µL per well). Cells were cultured for up to 96 h at 37 °C and 5% CO_2_ without medium change. All conditions were tested in triplicate.

Proliferation of N2a cells was determined based on the number of cells. For this purpose, high-power field images (HPFs) from the center of the well (×5 magnification) were taken from each well using a phase-contrast microscope after 48 h and 96 h. The cell-counting function of the IKOSA AI image analysis software (KOLAIDO, Altenrhein, Switzerland) was used to determine the number of cells from each HPF.

Viability was assessed using the Alamar Blue assay. In this assay, resazurin, the main component of Alamar Blue, is reduced by viable and metabolically active cells to resorufin, resulting in a color change that can be quantified by measuring optical density (OD). After 48 h and 96 h incubation, the spent medium in all groups was replaced with MEM 3% supplemented with one-tenth volume of a pre-dissolved 10× resazurin solution (Resazurin, Sigma-Aldrich, St. Louis, MO, USA). Cells were incubated for an additional 90 min at 37 °C. Subsequently, 100 µL of supernatant from each well was transferred to a 96-well plate, and fluorescence was measured at 560 nm using a microplate reader (Mithras LB 940, Berthold Technologies GmbH & Co. KG, Bad Wildbad, Germany).

Cytotoxicity was assessed by measuring lactate dehydrogenase (LDH) activity (LDH Cytotoxicity Assay Kit; Hoffmann-La Roche, Basel, Switzerland). After 48 h and 96 h, 150 µL of supernatant from each well was transferred to a 96-well plate. From these samples, 100 µL was transferred to new wells of the same plate, followed by the addition of 100 µL of catalyst solution. The plate was incubated in the dark for 30 min before fluorescence was measured at 490 nm using a microplate reader (Mithras LB 940, Berthold Technologies GmbH & Co. KG, Bad Wildbad, Germany).

### 2.8 Scratch assay

For scratch assay, cells were seeded into two-chamber culture inserts that create a 500-µm-wide gap (ibidi GmbH, Gräfelfing, Germany). N2a cells were suspended in basal medium and seeded into inserts mounted in 24-well plates at a density of 40,000 cells/cm^2^ (70 µL per chamber). To prevent cell desiccation, additional basal medium (350 µL per well) was added around the inserts. Cells were allowed to adhere for 48 h at 37 °C and 5% CO_2_ before the inserts were removed. The basal medium was then removed, and cells were cultured with experimental media consisting of HPS, NS, and PRP (0.1%, 1%, and 10%) as well as positive and negative controls (500 µL per well). Gap closure was documented at 0, 24, and 48 h using an inverted phase-contrast microscope. Quantitative analysis of the remaining cell-free area was subsequently performed using the image analysis tool of IKOSA AI Software (KOLAIDO, Altenrhein, Switzerland). Because no proliferation inhibitor was used, the scratch assay reflects combined migratory and proliferative wound closure rather than migration alone.

### 2.9 Neurite formation assay

For the neurite formation assay, N2a cells were seeded at a density of 5,000 cells in basal medium per well in 24-well plates and incubated for 24 h at 37 °C and 5% CO_2_ to allow adequate cell adhesion. After incubation, the basal medium was removed, and cells were cultured in the respective experimental media. A total volume of 1,000 µL of medium was used per well.

Neurite outgrowth was monitored using a phase-contrast microscope at 48 h and 96 h. At each time point, HPF images were acquired from all four quadrants of each well at ×40 magnification to comprehensively document neurite growth. Image analysis was subsequently performed using the IKOSA AI Software (KOLAIDO, Altenrhein, Switzerland) with the Neurite Outgrowth Analysis Tool, enabling quantitative assessment of neurite outgrowth in N2a cells. Parameters analyzed included the number of neurites, neurite area, neurite length, branching points, and number/percentage of neurite-bearing cells.

### 2.10 Statistical analysis

Data sets were analyzed using repeated measures of one-way analysis of variance (ANOVA), followed by subsequent comparisons using Tukey’s *post hoc* analysis. If two independent variables were present, a two-way repeated measures ANOVA with subsequent comparisons using Tukey’s *post hoc* analysis was performed. All values are expressed as means ± standard error of the mean (SEM). A value of p < 0.05 was considered statistically significant.

## 3 Results

### 3.1 Proteomic analysis of neurotrophic, regulatory, and inflammatory cytokines

**FIGURE 1:**
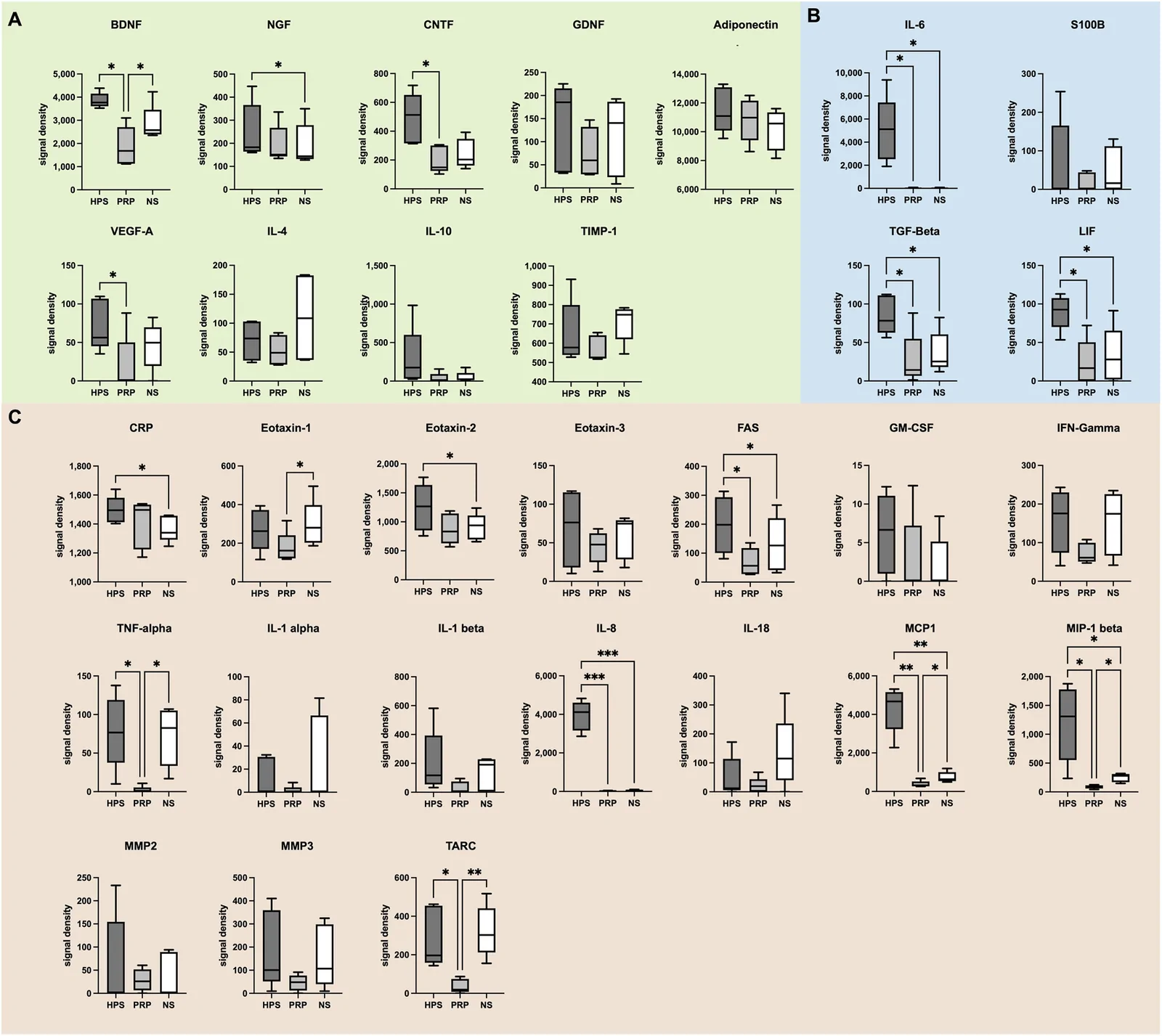
Semi-quantitative Human Neuro Discovery Array of HPS, PRP, and NS secretomes detecting 30 cytokines with **(A)** neurotrophic (green), **(B)** regulatory (blue), or **(C)** inflammatory functions (red). Measurements in signal density (arbitrary units). Boxplots displaying the 90th and 10th percentile at the whiskers, the 75th and 25th percentiles at the boxes and the median in the center line. One-way repeated-measures ANOVA with Tukey’s multiple comparison test. Data points are means ± SEM, blood donors: n = 5. *p < 0.05, **p < 0.01, ***p < 0.001.

As a first step in profiling the neurologically related cytokine composition of HPS and PRP, we performed a semi-quantitative proteomic array measuring 30 cytokines with neurotrophic, regulatory, or inflammatory functions, using normal serum (NS) as control (Figure 1). Among the regenerative cytokines (green; Figure 1A), HPS contained significantly higher levels of BDNF, CNTF, and VEGF-A compared to PRP, whereas PRP showed significantly lower BDNF levels than NS. NGF was detected at higher levels in HPS than in NS. Among the regulatory cytokines (blue; Figure 1B), HPS exhibited markedly higher concentrations of IL-6, TGF-β, and LIF than both PRP and NS, while S100B remained comparable across all groups. Regarding the neurodegenerative cytokines (red; Figure 1C), HPS showed elevated levels of CRP, Eotaxin-2, FAS, IL-8, MCP-1, and MIP-1β compared to NS. In contrast, PRP displayed significantly lower levels of FAS, TNF-α, IL-8, MCP-1, MIP-1β, and TARC relative to HPS.

### 3.2 Analysis of proliferation, viability, and cytotoxicity

**FIGURE 2:**
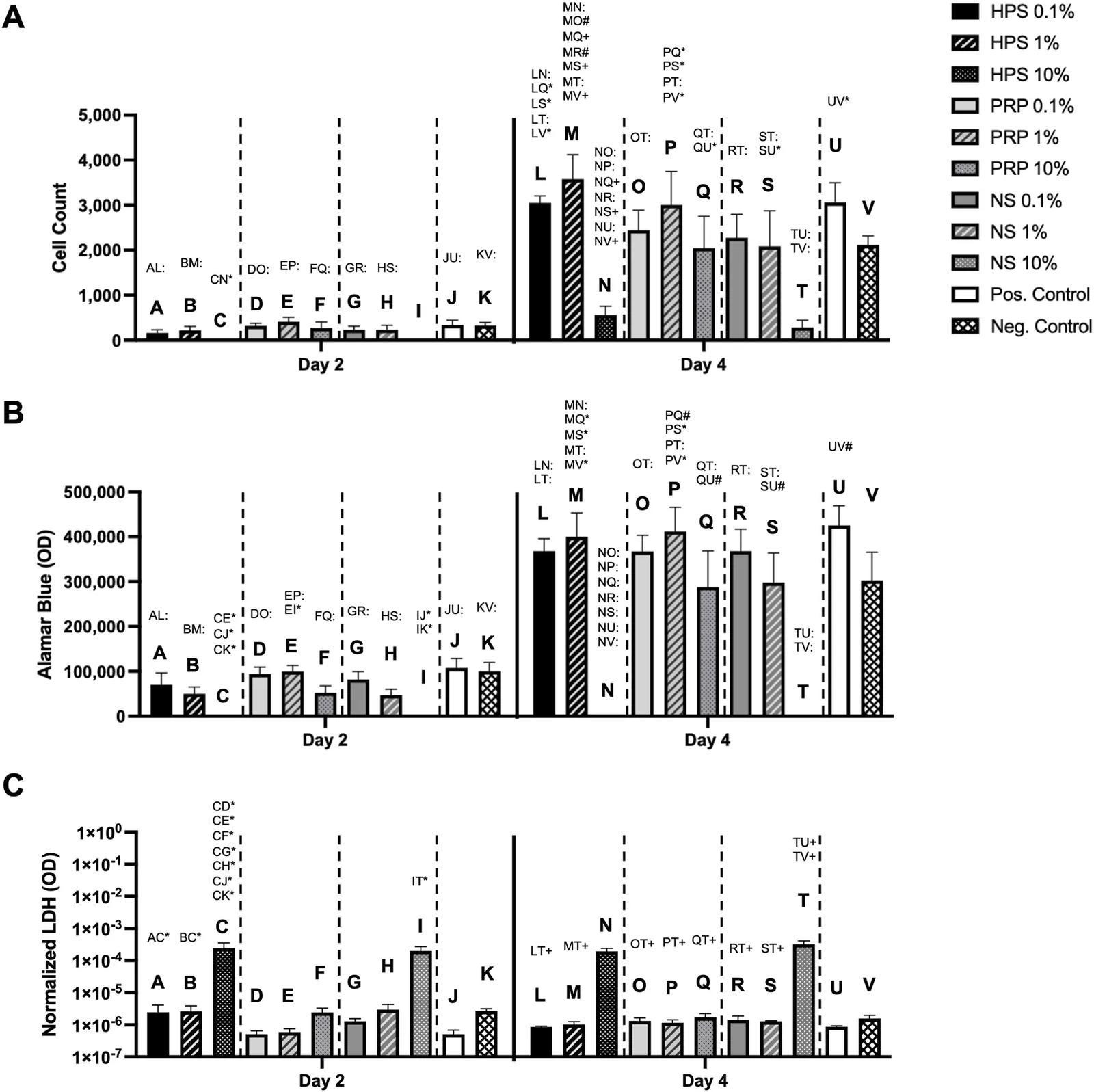
The effects of HPS, PRP, and NS on the proliferation, viability, and cytotoxicity of N2a cells. N2a cells were stimulated with HPS/NS/PRP-0.1%, −1%, −10%, compared to positive control (NGF) and negative control (basal medium). **(A)** Microscopic cell count of high-powered fields after 2 and 4 days of stimulation. **(B)** Alamar Blue assay: optical density (OD). **(C)** Lactate dehydrogenase (LDH) assay: normalized to viability value (Alamar Blue). Two-way repeated-measures ANOVA with Tukey’s multiple comparisons test. Data points are means ± SEM, results in triplicate. Capital letter pairs above the plots indicate statistical comparison of corresponding data points. For all pair comparisons, * = p < 0.05, # = p < 0.01, + = p < 0.001, : = p < 0.0001.

Proliferation, viability, and cytotoxicity of N2a cells were evaluated after 2 and 4 days of incubation with HPS and PRP at three secretome concentrations (0.1%, 1%, and 10%), and compared with NS as well as positive and negative controls. Cell proliferation was quantified by cell counting (Figure 2A). Significant increases in cell number from day 2 to day 4 were observed in all groups, except NS-10%, with the most pronounced effect detected in HPS-1% (16.3-fold increase; 219.7 vs. 3,577 cells, p < 0.0001). By day 4, cell numbers in the HPS-0.1% and HPS-1% groups were significantly higher than those in the negative control (up to 1.7-fold in HPS-1%: 3,577 vs. 2,114 cells, p = 0.003), while HPS-1% treated cells additionally exceeded the cell numbers of all NS concentrations. In contrast, both HPS-10% and NS-10% exhibited significantly lower cell counts compared with the negative control. Among PRP-treated groups, only PRP-1% demonstrated significantly higher cell numbers relative to the negative control (1.4-fold increase; 3,006 vs. 2,114 cells, p = 0.04).

Assessment of viability using the Alamar Blue assay revealed a comparable trend to the cell count analysis (Figure 2B). In particular, HPS-10% and NS-10% showed markedly reduced viability on day 4 compared with all other experimental conditions.

Cytotoxicity, determined by LDH release, demonstrated significantly elevated LDH levels in the HPS-10% group on day 2 and in the NS-10% group on day 4, with NS-10% additionally showing a significant increase in LDH release between day 2 and day 4. In contrast, all remaining treatment conditions exhibited low and comparable LDH levels, with no significant changes over time (Figure 2C).

### 3.3 Analysis of scratch closure activity

**FIGURE 3:**
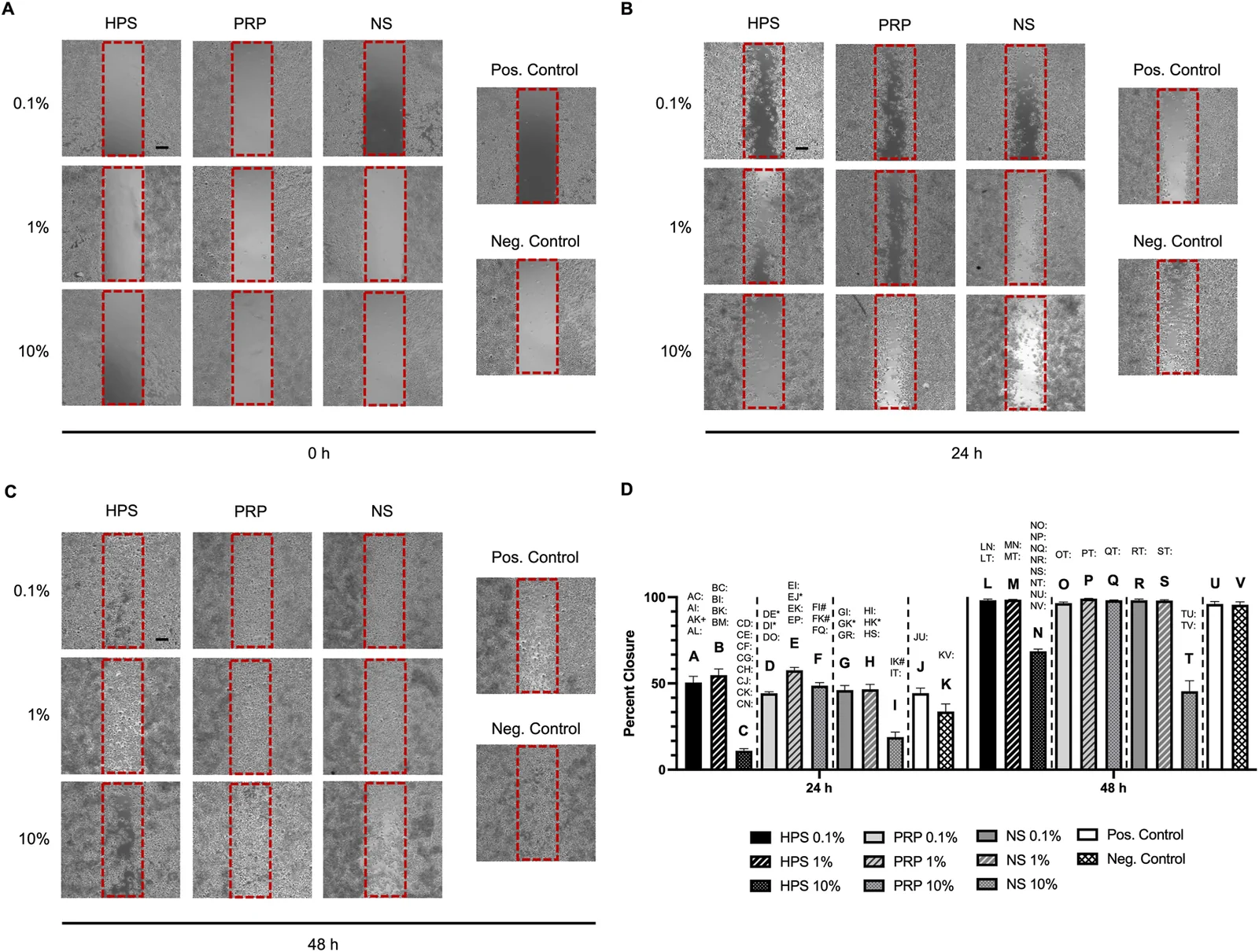
The effects of HPS, PRP, and NS on the scratch gap closure of N2a cells. **(A–C)** Representative microscopic photographs of the scratch assay with N2a cells stimulated with HPS/NS/PRP at concentrations of 0.1%, 1%, 10%, compared with positive control (NGF) and negative control (basal medium) taken at 0 h, 24 h and 48 h. Red boxes indicate the initially standardized open area. Scale bar = 200 μm. **(D)** Plot showing closure of the open area (in %) calculated by image analysis of the digital photographs shown in **(B,C)**. Two-way repeated-measures ANOVA with Tukey’s multiple comparisons test. Data points are means ± SEM, results in triplicate. Capital letter pairs above the plots indicate statistical comparison of corresponding data points. For all pair comparisons, * = p < 0.05, # = p < 0.01, + = p < 0.001, : = p < 0.0001.

The scratch assay evaluated the combined cell migration and proliferation capabilities of N2a neuroblastoma cells after 24 h and 48 h of secretome incubation (Figure 3). At 24 h, HPS/NS-0.1% and −1% as well as PRP-1% and −10% showed significantly greater gap closure compared with the negative control, with PRP-1% yielding the highest value (58%). In contrast, HPS-10% and NS-10% demonstrated markedly impaired gap closure relative to all other conditions. This pattern persisted at 48 h, with HPS-10% and NS-10% reaching only 45%–68% closure, whereas all other conditions achieved >96% closure.

### 3.4 Analysis of neurite formation

**FIGURE 4:**
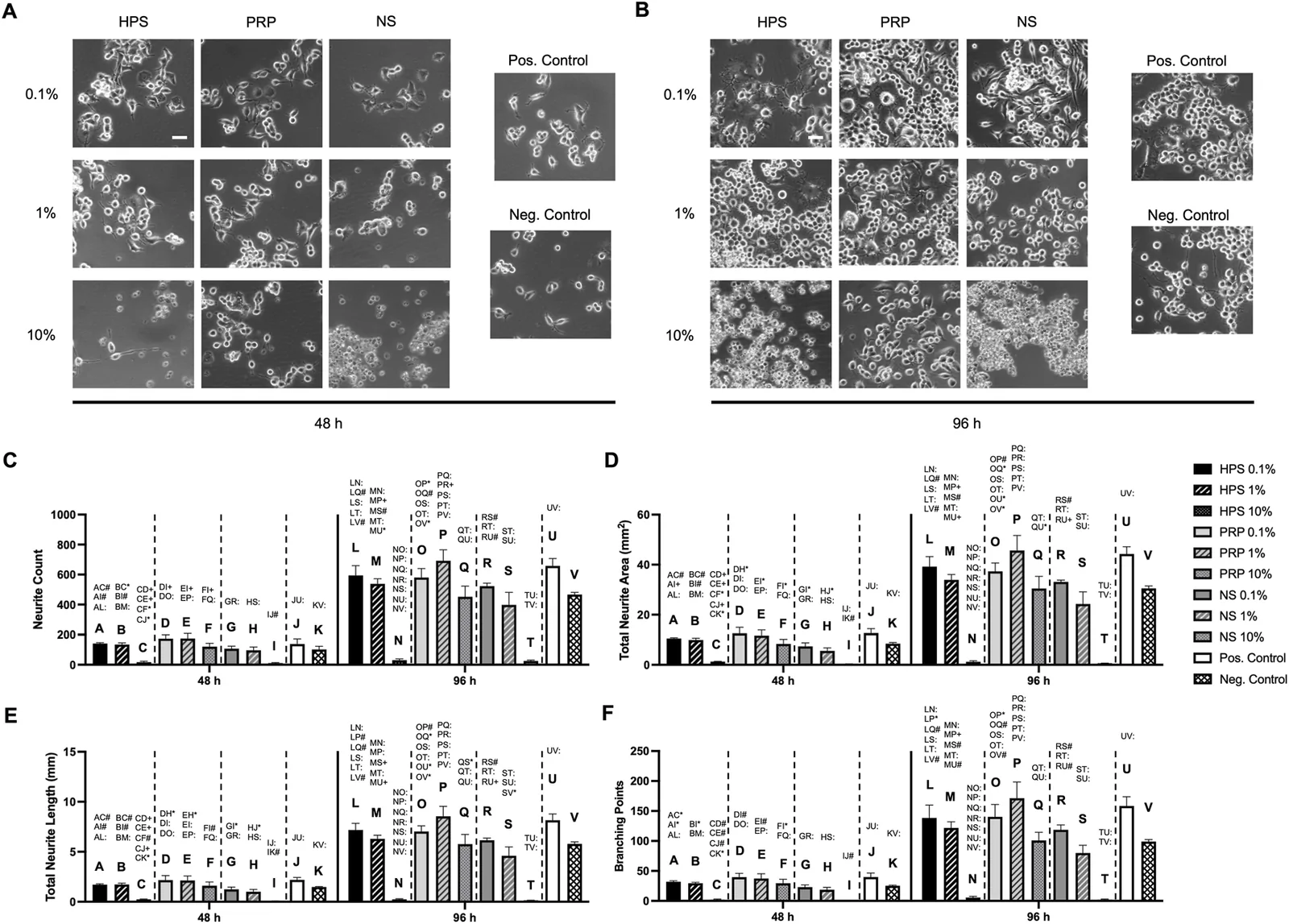
Effects of HPS, PRP, and NS on neurite network formation in N2a cells. Representative phase-contrast micrographs and quantitative analysis of neurite network formation following stimulation with HPS, PRP, or NS at concentrations of 0.1%, 1%, and 10%, compared with the positive control (NGF) and negative control (basal medium). **(A,B)** Representative micrographs acquired after 48 h **(A)** and 96 h **(B)** of incubation. Scale bar = 100 μm. **(C–F)** Quantitative image analysis of neurite network parameters: **(C)** total neurite count, **(D)** total neurite-covered area (mm^2^), **(E)** total neurite length (mm), and **(F)** total branching points. Two-way repeated-measures ANOVA with Tukey’s multiple comparisons test. Data points are means ± SEM, results in triplicate. Capital letter pairs above the plots indicate statistical comparison of corresponding data points. For all pair comparisons, * = p < 0.05, # = p < 0.01, + = p < 0.001, : = p < 0.0001.

Neurite outgrowth was assessed as a surrogate marker of neurogenic differentiation after 48 h (Figure 4A) and 96 h (Figure 4B) of secretome incubation. Differences in the overall neurite network became more pronounced at 96 h. With the exception of HPS-10% and NS-10%, all groups showed increased neurite count (Figure 4C), neurite-covered area (Figure 4D), total neurite length (Figure 4E), and number of branching points (Figure 4F) between 48 h and 96 h. Among all conditions, the lower concentrations of HPS and PRP (HPS-0.1% and PRP-0.1%/1%) achieved the highest values across all parameters, reaching levels comparable to the positive control. Compared with the negative control, values were up to 1.7-fold higher in PRP-1% (all p < 0.0001) and 1.4-fold higher in HPS-0.1% (all p < 0.01) across all parameters.

**FIGURE 5:**
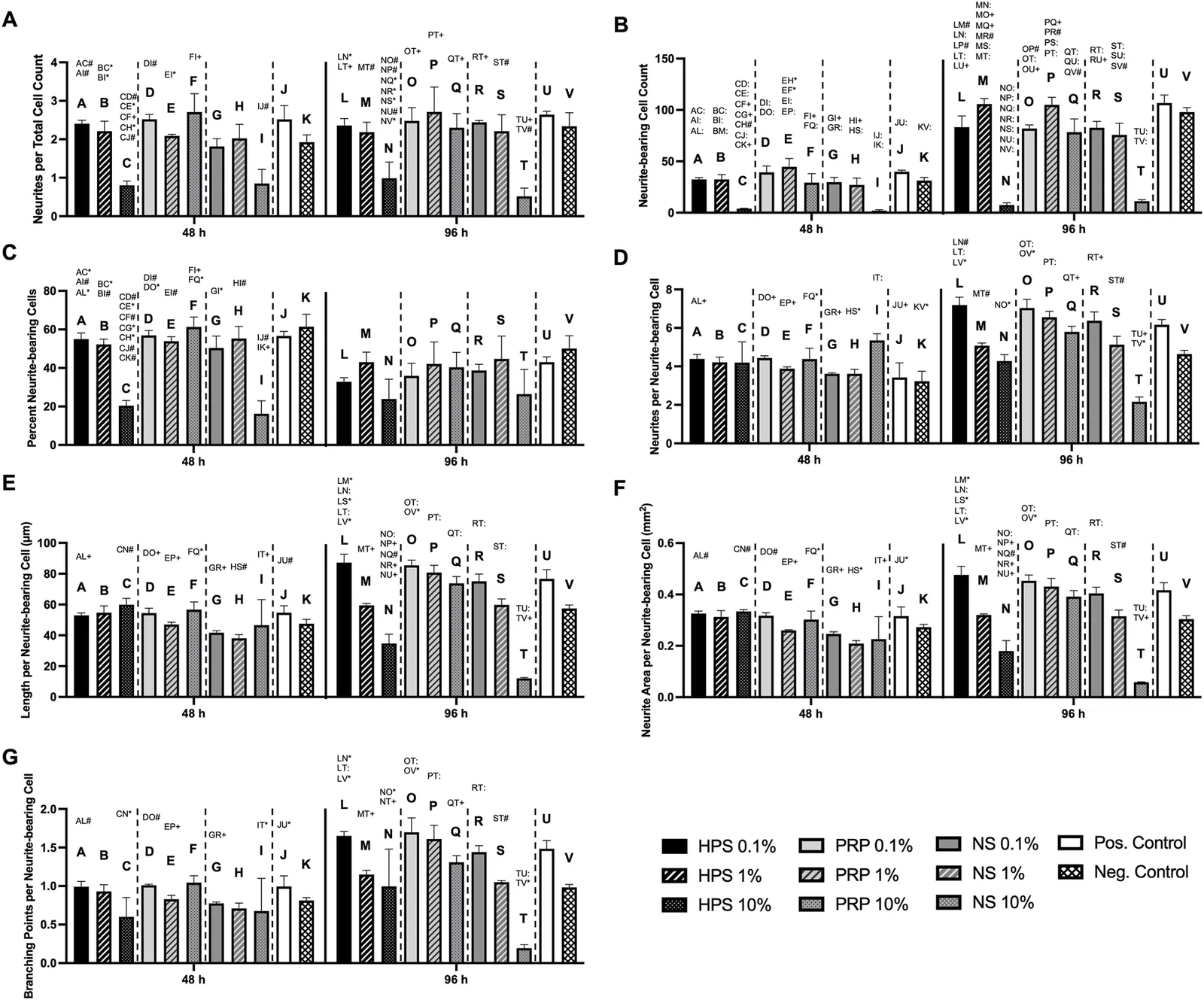
Normalization of neurite parameters to distinguish proliferation from neurite development. Quantitative image analysis of the micrographs depicted in Figures 4A,B. **(A)** Neurite count normalized to total cell count. **(B)** Absolute neurite-bearing cell count (NBCs). **(C)** Percentage of neurite-bearing cells relative to the total cell population. **(D)** Neurite count per NBC. **(E)** Neurite length per NBC (µm). **(F)** Neurite-covered area per NBC (mm^2^). **(G)** Branching points per NBC. Two-way repeated-measures ANOVA with Tukey’s multiple comparisons test. Data points are means ± SEM, results in triplicate. Capital letter pairs above the plots indicate statistical comparison of corresponding data points. For all pair comparisons, * = p < 0.05, # = p < 0.01, + = p < 0.001, : = p < 0.0001.

Notably, when normalizing neurite count to the cell number, no significant increase was observed between 48 h and 96 h (Figure 5A). At 96 h, the neurite per cell number ratio was comparable across all the conditions except HPS-10% and NS-10% which showed a lower ratio. Further visual inspection of representative micrographs revealed the coexistence of proliferative cell clusters and differentiated regions characterized by extensive neurite networks at 96 h (Figure 4B). To distinguish the effects of proliferation from neurite formation, the number of neurite-bearing cells (NBCs) was quantified (Figure 5B). The absolute number of NBCs increased from 48 h to 96 h in all treatment groups except for HPS-10% and NS-10%. However, when expressed as a percentage of the total cell population, the proportion of NBCs decreased about 20%-22% between 48 h and 96 h in HPS/PRP-0.1% and PRP-10% (p < 0.05), indicating that expansion of the non-NBCs outpaced the increase in NBCs in these conditions (Figure 5C). Normalization of neurite parameters to the number of NBCs revealed significant increases in neurite count (Figure 5D), total neurite length (Figure 5E), neurite-covered area (Figure 5F), and branching points per NBC (Figure 5G) between 48 h and 96 h in HPS-0.1% and all PRP conditions. At 96 h, these parameters were up to 1.7-fold higher in both HPS-0.1% and PRP-0.1%-treated cultures than in the negative control (p < 0.05).

## 4 Discussion

This study demonstrates that both Hypoxia Preconditioned Serum (HPS) and Platelet-Rich Plasma (PRP) exert concentration-dependent effects on N2a neuroblastoma cells, promoting proliferation, migration, and neurite network formation. By integrating proteomic profiling with functional assays, our findings position HPS and PRP as biologically active secretomes that support expansion of the overall neurite network while enhancing neurite elaboration within the neurite-bearing cell population.

Semiquantitative proteomic analysis revealed that HPS is enriched in several neurotrophic and angiogenic factors, including BDNF, CNTF, NGF, and VEGF-A. These factors are have been associated with neuronal survival, differentiation, and axonal growth, particularly under hypoxic or injury-associated conditions (Madduri et al., 2010; Su et al., 2020; Yang et al., 2011). Although PRP has been reported in the literature to contain BDNF, the concentrations detected in our study were lower than those in HPS (Malange et al., 2025; Ng, 2025). CNTF is predominantly produced by glial cells and, to a lesser extent, by T-cells; it is therefore not considered a major platelet-derived component, and its higher abundance in HPS likely reflects its leukocyte origin (Tormo et al., 2012). Platelets store NGF in their secretory granules, but the main sources of NGF are nervous system cells (glial cells, oligodendrocytes, neurons), structural cells (fibroblasts, keratinocytes, endothelial cells), and immune cells (mast cells, B-/T-cells), which is consistent with the higher NGF levels observed in the peripheral blood cell (PBC)-derived HPS (Wang et al., 2022; Kniewallner et al., 2014). VEGF-A, beyond its angiogenic role, exerts direct neurotrophic effects by enhancing neuronal survival and axonal outgrowth, thereby contributing to neurite elongation and structural stabilization during nerve regeneration (Sondell et al., 1999; Jin et al., 2006). Notably, our previous proteomic work identified significantly higher VEGF-A levels in HPS than in PRP, corroborating the current protein array results (Moog et al., 2020; Jiang et al., 2025). In addition to these neurotrophic mediators, HPS contained higher levels of regulatory cytokines such as IL-6, TGF-β, and LIF than PRP. These factors act as modulators of nerve repair rather than purely pro-regenerative signals: Together, they help initiate and coordinate inflammation, Schwann cell responses, and matrix remodeling, but excessive or dysregulated signaling of any of them may also constrain or distort regeneration (Rothaug et al., 2016; Li et al., 2017; Chen et al., 2021). At the same time, HPS exhibited higher concentrations of several cytokines typically associated with neurodegenerative or pro-inflammatory signaling (FAS, TNF-α, MCP-1, MIP-1β, and TARC) than PRP, implicating a possible reduction of pro-neurogenic and neuritogenic effect of HPS (Gonzalez Caldito, 2023; Yu and Fehlings, 2011; Conductier et al., 2010; Saika et al., 2012; Eivazitork et al., 2023). These cytokines characterize an inflammatory- and metabolically-stressed microenvironment in which hypoxia-induced signaling pathways are activated, consistent with the hypoxia-driven conditioning employed during HPS preparation (Lewis and Elks, 2019; Mogi et al., 2001; Mojsilovic-Petrovic et al., 2007; Zampetaki et al., 2004; Korbecki et al., 2020). Consequently, the biological activities of HPS and PRP are likely determined by the integrated action of both regenerative and inflammatory mediators rather than by individual cytokines alone. This concept is further supported by our functional experiments, in which HPS and PRP produced comparable biological responses despite their substantially different cytokine compositions. Because the Human Neuro Discovery Array provides semiquantitative protein profiling, the present data should be interpreted as descriptive rather than mechanistic. Future studies employing quantitative proteomics together with functional approaches, such as neutralizing antibodies or pathway inhibition, will be required to identify the specific factors and signaling pathways responsible for the observed effects.

Functionally, HPS significantly enhanced N2a cell proliferation, viability and scratch gap closure at low to intermediate concentrations (0.1%–1%). In contrast, the highest HPS concentration (10%) was associated with reduced cell viability and gap closure activity, indicating a dose-dependent decline in biological activity. This biphasic response mirrors previous HPS studies in angiogenesis, lymphangiogenesis, and cartilage repair, where concentration-dependent increases in cellular proliferation, migration, and differentiation were most pronounced within a specific concentration range and diminished at higher concentrations (Jiang et al., 2023a; Jiang et al., 2023b; Moog et al., 2020). These results also indicate that HPS should not be regarded as a universal growth factor supplement but rather as a tightly regulated biological stimulus whose efficacy depends critically on precise dosing. In the context of peripheral nerve repair, these responses should be interpreted as surrogate markers of neuronal plasticity, cytoskeletal remodeling, and a growth-oriented regenerative phenotype (Khodosevich et al., 2009; Wesley et al., 2015). Conversely, PRP maintained proliferative and gap closure activity also at higher concentrations (PRP-10%), with PRP-1% exhibiting the highest values. This broader effective concentration range may reflect differences in the composition and balance of trophic and inflammatory mediators between PRP and HPS, although the mechanisms underlying these differences remain to be determined. It must be noted that the scratch assay cannot distinguish between true migration and proliferation-associated gap closure. Therefore, it should be interpreted as combined proliferative and migratory activity.

Further effects of HPS and PRP on N2a cells were identified in the neurite formation assay. Treatment with HPS (0.1%) and PRP (0.1% and 1%) resulted in marked increases in total neurite count, neurite length, neurite-covered area, and branching points, reaching levels comparable to the positive control and significantly exceeding those of the negative control after 96 h. However, normalization of neurite count to the total cell number abolished these differences, indicating that the expansion of the overall neurite network occurred in parallel with the pronounced proliferative response induced by both secretomes. To further distinguish the effects of proliferation from neurite development, neurite-bearing cells (NBCs) were quantified. Although the absolute number of NBCs increased over time, their proportion relative to the total cell population declined, indicating that expansion of the non-NBC population outpaced the increase in NBCs. Interestingly, normalization of neurite parameters to the number of NBCs revealed significant increases in neurite count, neurite length, neurite-covered area, and branching points per NBC. Notably, the enhanced neurite elaboration was predominantly observed at low secretome concentrations: HPS-0.1% consistently produced the strongest effects among all HPS concentrations, whereas PRP-0.1% (and to a lesser extent PRP-1%) yielded the highest neurite count, neurite length, neurite-covered area, and branching points per NBC. In contrast, the highest concentration (10%) was consistently less effective. The observation that lower concentrations consistently outperformed higher concentrations suggests that the neurite network-forming activity of both HPS and PRP follows a biphasic dose-response relationship here as well. Comparable biphasic responses have been described for numerous growth factors (e.g., NGF, BDNF) where moderate concentrations optimize intracellular signaling, whereas excessive stimulation may activate negative feedback mechanisms or expose cells to higher concentrations of inhibitory mediators (Calabrese, 2008; Conti et al., 2004). Together with the microscopic observation of proliferative cell clusters adjacent to differentiated regions exhibiting extensive neurite networks, these findings suggest that HPS and PRP simultaneously promote expansion of the overall cell population while enhancing neurite elaboration within the differentiated cell subpopulation. Similar dual effects of HPS on proliferation and lineage-specific differentiation have previously been reported in chondrocytes, osteoblasts, and preadipocytes (Jiang et al., 2023a; Jiang et al., 2022b; Jiang et al., 2025). Although the present data do not demonstrate increased neuronal differentiation *per se*, they indicate that both HPS and PRP support morphological maturation of NBCs despite the predominance of proliferative responses. To our knowledge, this study provides the first evidence that HPS enhances neurite network formation and neurite elaboration in an established neuronal differentiation model. While PRP has previously been shown to stimulate neurite outgrowth in neuronal explant systems, its effects have not yet been investigated in standardized neurite differentiation models such as N2a or PC12 cells. In spiral ganglion neuron cultures, human PRP significantly increased neuronal survival and neurite length in a dose-dependent manner, an effect that was reversed when endogenous neurotrophic signaling was blocked, underscoring the dependence of PRP-induced neurite extension on classical neurotrophic pathways (Stolle et al., 2018). Likewise, treatment of human dental pulp stem cells with PRP resulted in enhanced neurogenic differentiation and formation of βIII-tubulin-positive neurons with neurite-like processes, accompanied by reduced apoptosis both *in vitro* and in a rat spinal cord injury model (Hu et al., 2022). Collectively, these observations are consistent with our findings that HPS- and PRP-derived secretomes promote expansion of the overall neurite network while enhancing neurite elaboration within differentiated cells. Future studies employing primary dorsal root ganglion cultures and *in vivo* peripheral nerve injury models will be required to determine whether these morphological changes translate into enhanced axonal elongation and functional nerve regeneration.

Our findings also fit into the broader context of hypoxia-based and growth factor-driven strategies for peripheral nerve repair. Hypoxic preconditioning of Schwann-like cells, neural progenitors, or adipose-derived stem/progenitor cells has been shown to enhance their resistance to hypoxic stress and to increase the secretion of neuroprotective and angiogenic factors such as BDNF, NGF, GDNF, and VEGF, which in turn improves axonal regrowth and functional recovery when these cells are delivered within nerve conduits or grafts (Mayer et al., 2023; Sumarwoto et al., 2023; Chen et al., 2015). Parallel work on growth-factor supplementation has demonstrated that controlled delivery of multiple growth factors (e.g., NGF, GDNF, BDNF, CNTF, and VEGF) via biomaterials can more effectively promote Schwann cell migration, axonal extension, remyelination, and target reinnervation than single-factor approaches, particularly when release is spatially and temporally regulated (Li et al., 2020). In this light, HPS can be viewed as a cell-free, hypoxia-driven autologous cocktail that provides a physiological combination of neurotrophic and angiogenic mediators, conceptually similar to combining several recombinant growth factors within an advanced nerve conduit but with simpler preparation and potentially more favorable regulatory and cost profiles. The strong effect of low-dose HPS on neurite elaboration observed in our study suggests that such hypoxia-conditioned blood-derived secretomes could be integrated into next-generation nerve guidance conduits or hydrogel systems to leverage their multifactorial signaling while benefiting from established biomaterial delivery platforms. PRP has been extensively investigated as an adjunct therapy in peripheral nerve repair, with recent reviews summarizing its multifaceted neuroprotective and neuroregenerative actions in preclinical and clinical settings (Wang et al., 2022). Collectively, these studies suggest that PRP promotes nerve regeneration through multiple complementary mechanisms, including protection against neuronal apoptosis, stimulation of neovascularization, promotion of axonal regrowth and remyelination via Schwann cell activation, and modulation of the inflammatory microenvironment, all of which contribute to improved sensory and motor recovery and reduction of neuropathic pain (Shang et al., 2025; Zhu et al., 2025; Zheng et al., 2016). Experimental studies support these observations at multiple levels. *In vitro*, moderate PRP concentrations (2.5%–20%) maximally stimulated Schwann cell proliferation, migration, and NGF/GDNF expression, whereas higher concentrations attenuated these responses, indicating a concentration-dependent biological effect (Zheng et al., 2016). *In vivo*, a rabbit sciatic nerve crush model demonstrated that serial, ultrasound-guided PRP injections significantly accelerated electrophysiological and functional recovery (Zhu et al., 2025). Other experimental studies and systematic evaluations likewise report enhanced axonal growth and reduced scarring when PRP is applied around repaired or grafted nerves, and recent work has also explored PRP-derived exosomes and PRP-loaded biomaterials to further enhance regeneration (Wang et al., 2024; Zhang et al., 2024). Within this established framework, our findings extend the current literature by providing the first direct comparison of HPS and PRP in a standardized N2a neurite differentiation model.

Several limitations should be acknowledged. First, the use of N2a neuroblastoma cells limits direct translation to mature peripheral neurons and does not reproduce the cellular complexity of peripheral nerve regeneration. Second, the study combines human-derived secretomes with a murine cell line, introducing an interspecies component that may affect responsiveness. Third, the secretomes were pooled before use, which improves standardization but reduces visibility of donor-to-donor variability. Fourth, the proteomic array was semiquantitative and should therefore be interpreted as a profiling tool rather than an absolute measurement of factor concentration. Fifth, NS served as a clinically relevant serum comparator but differs from HPS not only in oxygen exposure, but also in incubation duration, coagulation, leukocyte activation, and accumulation of secreted mediators. Therefore, comparisons between HPS and NS reflect the combined effects of the HPS preparation process rather than the isolated contribution of hypoxic conditioning alone. Despite these limitations, the consistent concentration-dependent effects observed across multiple functional assays support the biological activity of HPS and PRP and their capacity to enhance neurite network formation and neurite elaboration in an *in vitro* neuronal model. Future studies should validate these findings in primary neuronal and Schwann cell co-culture systems, as well as in relevant *in vivo* models of peripheral nerve injury.

## Data Availability

The raw data supporting the conclusions of this article will be made available by the authors, without undue reservation.
